# Triterpenoid Saponins from *Stauntonia chinensis* Ameliorate Insulin Resistance via the AMP-Activated Protein Kinase and IR/IRS-1/PI3K/Akt Pathways in Insulin-Resistant HepG2 Cells

**DOI:** 10.3390/ijms150610446

**Published:** 2014-06-10

**Authors:** Xin Hu, Sha Wang, Jing Xu, De-Bing Wang, Yu Chen, Guang-Zhong Yang

**Affiliations:** 1 Laboratory for Natural Products Chemistry, College of Pharmacy, South Central University for Nationalities, Wuhan 430074, China; E-Mails: huxin5540@126.com (X.H.); shaqueen@sina.com (S.W.); xuj@mail.scuec.edu.cn (J.X.); biner70@sina.com (D.-B.W.); 2 College of Chemistry and Material Sciences, South Central University for Nationalities, Wuhan 430074, China; E-Mail: chenyuwh888@126.com

**Keywords:** *Stauntonia chinensis*, insulin-resistance, AMPK, IR/IRS-1/PI3K/Akt signaling pathway, HepG2 cells

## Abstract

Inflammation and oxidative stress play crucial roles in the etiology of type 2 diabetes mellitus. In this study, we examined the anti-diabetic effects of triterpenoid saponins extracted from *Stauntonia chinensis* on stimulating glucose uptake by insulin-resistant human HepG2 cells. The results showed that saponin **6** significantly increased glucose uptake and glucose catabolism. Saponin **6** also enhanced the phosphorylation of AMP-activated protein kinase (AMPK) and activated the insulin receptor (IR)/insulin receptor substrate-1 (IRS-1)/phosphoinositide 3-kinase (PI3K)/Akt pathway. Therefore, our results suggest that saponins from *S. chinensis* improve glucose uptake and catabolism in hepatic cells by stimulating the AMPK and the IR/IRS-1/PI3K/Akt signaling pathways. The results also imply that saponins from *S. chinensis* can enhance glucose uptake and insulin sensitivity, representing a promising treatment for type 2 diabetes mellitus.

## 1. Introduction

Type-2 diabetes mellitus (T2DM) is a chronic metabolic disease in modern society that will affect over 366 million people worldwide by the year 2025 [1]. Insulin resistance is the main pathogenic event in T2DM and is characterized by a failure of tissues to respond to insulin, leading to a reduction in glucose uptake by peripheral tissue and increased hepatic glucose output [2,3]. Many studies have highlighted the importance of achieving optimal glucose control through strict adherence to medications, diet, and exercise to reduce the risk of serious long-term complications [4]. Because existing antidiabetic agents are often associated with side effects, there is increasing interest in the use of natural products for pharmacological purposes, to complement or replace existing therapies. To date, researchers have reported that a variety of natural products derived from medicinal herbs exhibit hypoglycemic properties, particularly triterpenes, flavonoids, and polyphenols [5].

AMP-activated protein kinase (AMPK) is a serine/threonine kinase that is the main cellular regulator of glucose metabolism, including muscle glucose uptake [6], the expression of cAMP-stimulated gluconeogenic enzymes, such as phosphoenolpyruvate carboxykinase (PEPCK) and glucose 6-phosphatase (G6Pase) [7], and the expression of glucose-stimulated genes associated with hepatic lipogenesis, such as fatty acid synthase, Spot-14, and L-type pyruvate kinase (PK) [8]. Therefore, AMPK plays a major regulatory role in metabolic disorders, such as diabetes, obesity, and cancer. AMPK was also reported to enhance fatty acid oxidation and decrease the production of glucose, cholesterol, and triglycerides in the liver following activation by an increase in the AMP:ATP ratio [9], exercise, increases in hormones levels (e.g., adiponectin and leptin), and intracellular stress (e.g., glucose deficiency, hypoxia, and oxidative stress) [10]. Thus, AMPK is a candidate therapeutic target for T2DM. Other possible targets include the insulin receptor (IR), which initiates a wide range of metabolic effects via its intrinsic tyrosine kinase. Activation of the IR tyrosine kinase causes phosphorylation on the tyrosine residues in a variety of docking proteins including members of the insulin receptor substrate (IRS) family [11].

Members of the IRS family also play important roles in IR signal transduction. Phosphorylated IRS proteins serve as multisite docking proteins for various effector molecules possessing src homology 2 (SH2) domains, including phosphatidylinositol 3-kinase (PI3K), regulatory subunits (p85, p55 p50, p85, and p55PIK), the tyrosine kinases Fyn and Csk, the tyrosine protein phosphatase SHP-2/Syp, and several smaller adapter molecules such as growth factor receptor binding proteins (e.g., Grb-2, Crk, and Nck) [12]. Activated IRS-1 phosphorylates the p85 regulatory subunit PI3K, which phosphorylates downstream protein kinase B (PKB/Akt) via its pleckstrin homology domain, leading to the phosphorylation of serine 307 (Ser 307), which ultimately stimulates glucose uptake in the liver. However, phosphorylation of IRS-1 at Ser 307 also inhibits the interaction between the phosphotyrosine binding domain of IRS-1 with the phosphorylated NPEY motif in the activated IR, disrupting insulin signaling, and may ultimately cause insulin resistance [13]. The AMPK and IR/IRS-1/PI3-K/Akt pathways are therefore critical signaling pathways in the regulation of glucose metabolism, and appear to contribute to the development of insulin resistance.

*Stauntonia chinensis* DC., also known as “Ye Mu Gua”, has been used as a traditional herbal medicine because of its anti-inflammatory and analgesic properties [14]. Previous phytochemical investigations revealed that this herb contained a number of triterpenoid saponins [15,16,17,18,19,20,21,22,23]. Acid hydrolysis of the triterpenoid glycosides of yielded three major aglycones: hederagenin, 30-norhederagenin and abkebonic acid. 30-Norhederagenin exhibited anti-diabetic properties by increasing the levels of phosphorylated (p)-AMPK, *p*-acetyl-CoA carboxylase, and *p*-glycogen synthase kinase-3b, and triggered glucose uptake and glycogen synthesis in insulin-resistant human hepatocellular carcinoma cells (HepG2) cells [24]. However, the mechanisms underlying the antidiabetic effects of triterpenoid saponins isolated from *S. chinensis* remain unclear.

**Figure 1:**
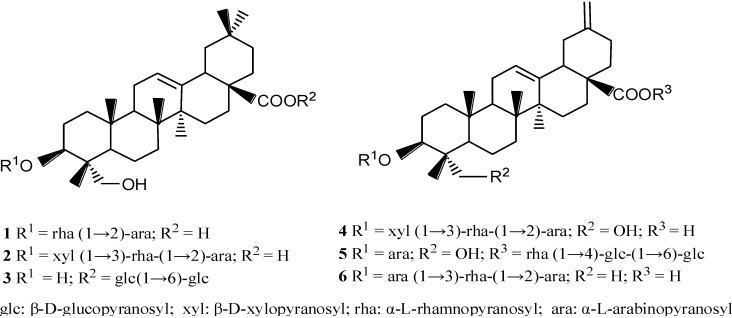
Structures of compounds **1**–**6** extracted from *S. chinensis*.

On the basis of these findings, we focused on the isolation of triterpenoid saponins from *S. chinensis* as potential anti-diabetic agents. In our previous studies examining the chemical constituents of *S. chinensis*, we isolated 10 triterpenoid saponins, including two new saponins (yemuoside YM_36–37_) [25]. In the present work, and in our prior research of triterpenoid saponins, we investigated the anti-diabetic properties of six triterpenoid saponins (Figure 1) from *S. chinensis* in insulin-resistant HepG2 cells. HepG2 cells were used in this study as a model of insulin-resistance because their bioactivities are similar to normal hepatic cells [26]. Insulin-resistance in HepG2 cells is principally associated with deficient glycogen synthesis and failure to suppress glucose production. Therefore, we examined the effects of triterpenoid saponins on the activities of the AMPK, IR/IRS-1/PI3K/Akt signaling pathways in an *in vitro* model of hepatic insulin resistance.

## 2. Results

### 2.1. Cytotoxicities of Compounds 1–6 and Their Effects on Glucose Uptake in Insulin-Resistant HepG2 Cells

**Figure 2:**
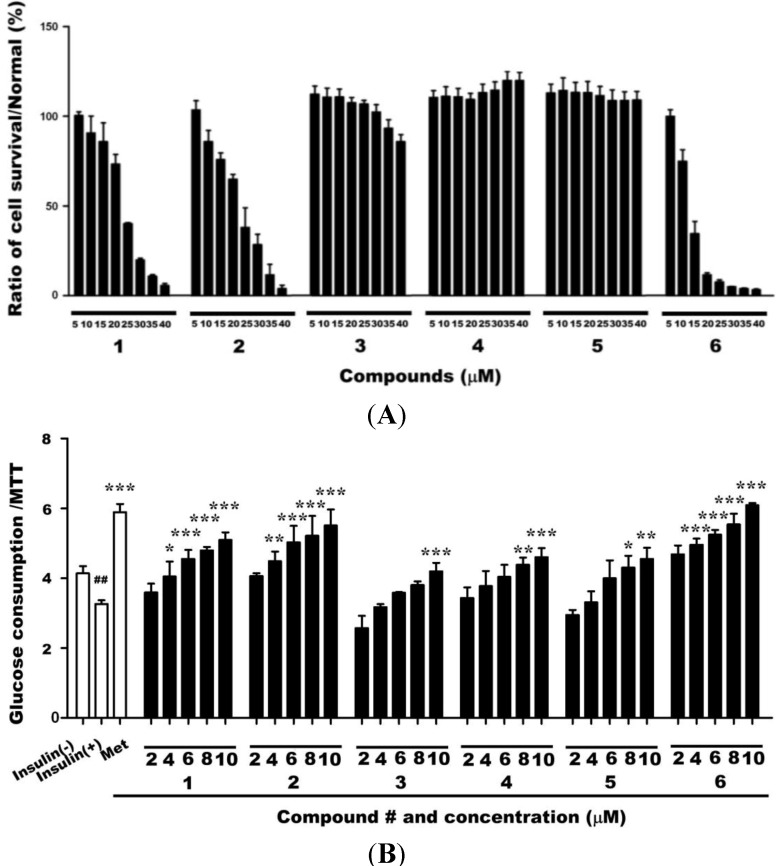
(**A**) Exposure to compounds **1**–**6** at concentrations of <10 µm for 24 h did not reduce the survival of HepG2 cells; (**B**) Exposure to 2–10 µm of compounds **1**–**6** dose-dependently enhanced glucose uptake in insulin-resistant HepG2 cells. Data are presented as the mean ± SD of glucose uptake divided by the optical density determined in the MTT (*n* = 3). Met: metformin; 1–6: compounds **1**–**6**. ^#^ *p* < 0.05 and ^##^ *p* < 0.01 *versus* control cells in the absence of insulin (insulin(−));* *p* < 0.05, ** *p* < 0.01, and *** *p* < 0.001 *versus* control cells in the presence of insulin (insulin(+)).

To examine the cytotoxicities of compounds **1**–**6**, HepG2 cells were exposed to various concentrations of these compounds (5–40 µm) in serum-free cell culture medium for 24 h, followed by 3-(4,5-dimethylthiazol-2-yl)-2,5-diphenyltetrazoliumbromide (MTT) assays. Exposure to these saponins at concentrations of <10 µm did not reduce the survival of HepG2 cells (Figure 2A). We next treated insulin-resistant HepG2 cells with compounds **1**–**6** at concentrations if (2, 4, 6, 8, or 10 µm ) for 24 h to investigate the effects on glucose uptake by insulin-resistant HepG2 cells in a dose-dependent manner (Figure 2B) compared with untreated cells model (3.26 ± 0.11). Compound **6** induced the greatest increase in glucose uptake (6.09 ± 0.06 at 10 µm) followed by compounds **1**, **2**, **4**, and **5**. The increase in glucose uptake induced by compound **6** was also greater than that induced by metformin (5.8 ± 0.23).

### 2.2. Effects of Compound 6 on Hexokinase (HK) and PK Activities in Insulin-Resistant HepG2

**Figure 3:**
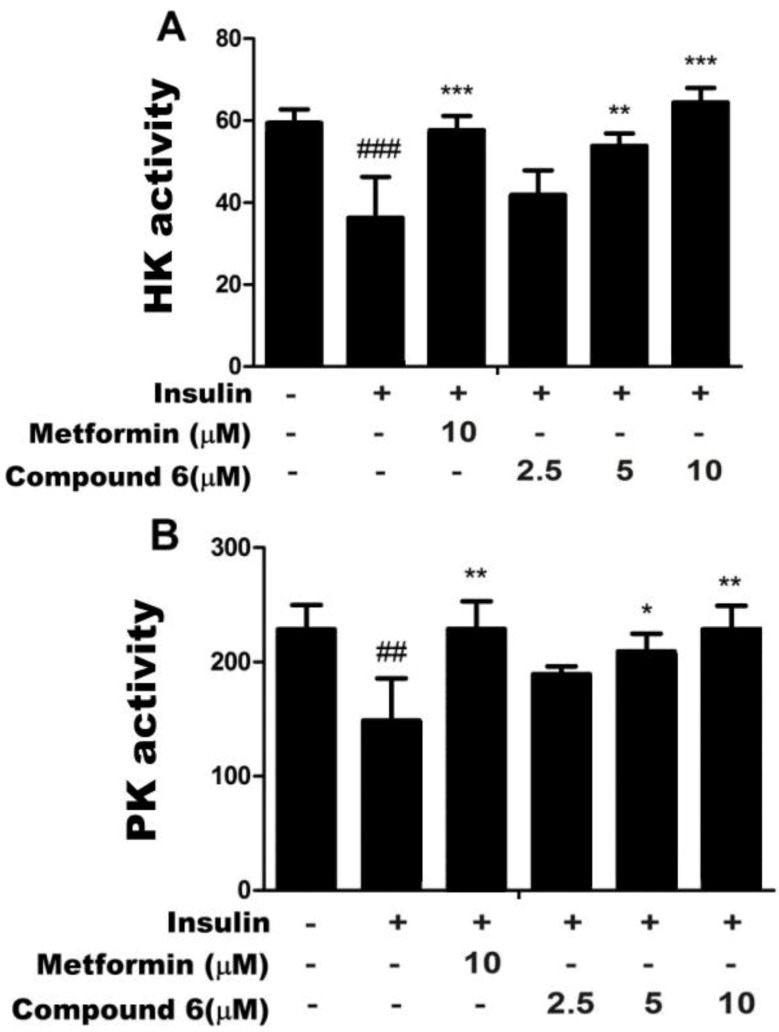
Compound **6** increased activities of hexokinase (HK) (**A**) and pyruvate kinase (PK); (**B**) in insulin-resistant HepG2 cells. Enzyme activity assays were performed to determine the activities of both enzymes. ^##^*p* < 0.01 and ^###^ *p* < 0.001 *versus* control cells, in the absence of insulin; * *p* < 0.05, ** *p* < 0.01, and *** *p* < 0.001 *versus* control cells in the presence of insulin.

Hexokinase (HK) and PK are key enzymes involved in glucose metabolism. HK is the key, rate-limiting enzyme in the glycolysis and glycogen synthesis pathways. PK is another key enzyme in glycolysis that catalyzes the transfer of a phosphate molecule from phosphoenolpyruvate to ADP, yielding pyruvate. Therefore, both enzymes are critical mediators of glucose metabolism, including glucose catabolism. Some reports suggest that the activities of several enzymes involved in glucose metabolism, including G6Pase, PEPCK, glycogen synthase, HK, and PK, are decreased in insulin resistance [26]. Therefore, we treated insulin-resistant HepG2 cell with 2.5, 5.0, or 10 µm of compound **6** to assess its effects on **6** HK and PK activities and insulin resistance. We found that compound **6** dose-dependently increased the activities of HK and PK (Figure 3). Furthermore, the increase in HK activity in cells treated with 10 µm of compound **6** was greater than in cells treated with metformin. Compound **6** restored the activities of enzymes in insulin-resistant HepG2 cells to levels similar to those in normal cells. Taken together, these results indicate that compound **6** may promote glucose metabolism, including glycogen synthesis and glycolysis, by enhancing HK and PK activities, and might therefore decrease blood-glucose levels.

### 2.3. Compound 6 Activates AMPK Phosphorylation in Insulin-Resistant HepG Cells

**Figure 4:**
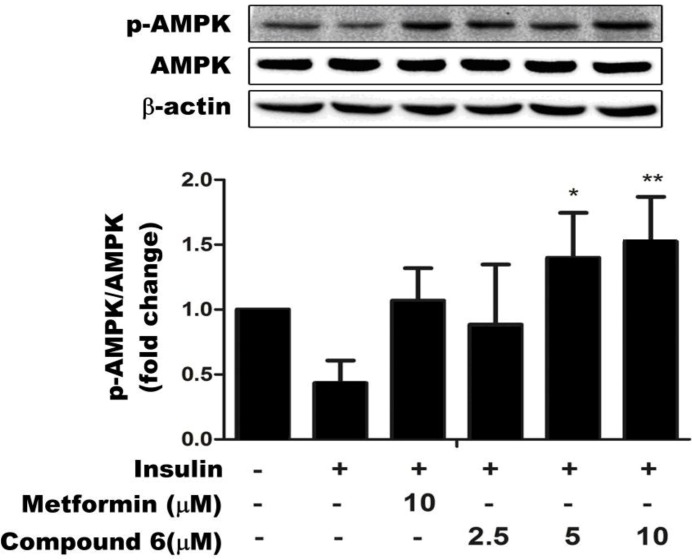
Compound **6** stimulates AMPK phosphorylation in a dose-dependent manner. Data are representative of three independent experiments. * *p* < 0.05 and ** *p* < 0.01 *versus* control cells in the presence of insulin.

AMPK is the main sensor of the cellular energy states, and plays an important role in regulating glucose metabolism. Therefore, it is considered to be a major target for the prevention and treatment of T2DM [27]. As shown in Figure 4, treatment with 2.5, 5, or 10 µm of compound **6** for 24 h dose- dependently increased the abundance of p-AMPK, without affecting the expression level of total AMPK (phosphorylated and unphosphorylated protein). Metformin also increased the abundance of p-AMPK. These results suggest that compound **6** regulates glucose metabolism in insulin-resistant HepG2 cells by enhancing AMPK activity.

### 2.4. Effects of Compound 6 on the IR/IRS-1/PI3K/Akt Signaling Pathway

**Figure 5:**
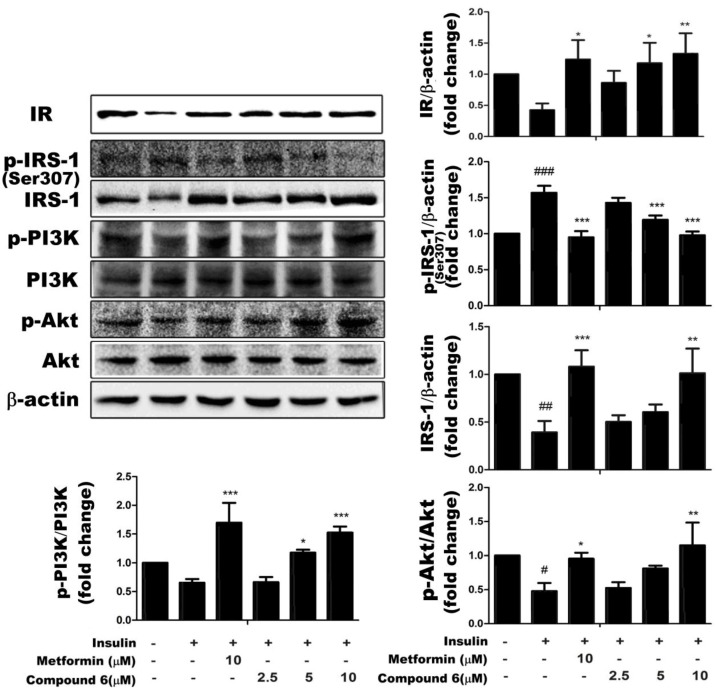
Compound **6** restored the activity of the IR/IRS-1/PI3K/Akt signaling pathway in HepG2/IR cells. HepG2/IR cell were treated with 0, 2.5, 5, 10 μm of compound **6** for the indicated times. Compound **6** increased the expression of insulin receptor (IR) expression, reduced IRS-1 phosphorylation at Ser 307, and stimulated the phosphorylation of PI3K and Akt compared with untreated cells. Data are presented as the mean of three independent experiments. ^#^ *p* < 0.05, ^##^ *p* < 0.01, and ^###^ *p* < 0.001 *versus* control cells in the absence of insulin; * *p* < 0.05, ** *p* < 0.01, and *** *p* < 0.001 *versus* control cells in the presence of insulin.

To determine the molecular mechanism underlying the effects of compound **6** on glucose metabolism, we determined the expression levels of proteins involved in the insulin signaling pathway by western blotting. As shown in Figure 5, compound **6** dose-dependently increased the expression of IR in insulin-resistant HepG2 cells. We then examined whether the degradation of IRS occurred in insulin-resistant HepG2 cells. Compound **6** dose-dependently reduced p-IRS-1 expression and increased total IRS-1 levels towards the levels observed in control insulin-sensitive cells. Compound **6** also significantly and dose-dependently increased the relative abundances of p-Akt and, p-PI3K without affecting the expression levels of total Akt or total PI3K. Metformin was used in these experiments as a positive control. These findings indicate that compound **6** increased the expression of IR, reduced p-IRS-1 levels, and activated the downstream PI3K/Akt signaling pathway, and therefore, enhanced insulin sensitivity in insulin-resistant HepG2 cells.

## 3. Discussion

The present study investigated the molecular mechanisms by which triterpenoid saponins enhance insulin sensitivity in insulin resistant HepG2 cells as a model of liver cells. Our results indicate that triterpenoid saponins from *S. chinensis*, especially compound **6**, significantly ameliorate insulin resistance by restoring the activities of HK and PK to the levels observed in control insulin-sensitive cells. Further novel findings of the present study are that triterpenoid saponins beneficially affect insulin sensitivity by activating the AMPK and IR/IRS-1/PI3K/Akt signaling pathways in insulin-resistant HepG2 cells.

Therapeutic interventions are needed to improve insulin sensitivity because insulin resistance eventually leads to T2DM. Previous studies showed that triterpenoid saponins from *S. chinensis* DC. has anti-inflammatory effects, which included significantly reduced release of nitric oxide and interleukin-1β from lipopolysaccharide-stimulated RAW264.7 cells [28]. Triterpenoid saponins from *Aralia elata* and *Astragalus* were also reported to have hypoglycemic effects [29,30]. Therefore, we hypothesized that triterpenoid saponins from *S. chinensis* DC., which exhibit potent anti-inflammatory and antioxidant effects, may also have antidiabetic effects. In our study, the compound **6** saponin, extracted from *S. chinensis*, increased HK and PK activities in insulin-resistant HepG2 cells towards the levels observed in control insulin-sensitive cells. These results indicated that compound **6** accelerates glucose metabolism in insulin-resistant cells, and may reduce blood-glucose levels *in vivo*.

Changes in IR expression, binding, phosphorylation, and/or tyrosine kinase activity could contribute to insulin-resistance [31]. Binding of insulin to its receptor induces the phosphorylation of tyrosine residues in proximal substrates, including members of the IRS family (IRS-1, -2, -3, and -4). IRS proteins are crucial signaling molecules that mediate the metabolic effects of insulin [32]. In hepatic insulin resistance, the expression of IR and the rate of IRS degradation are markedly reduced. As shown in Figure 5, compound **6** increased IR expression and enhanced the dephosphorylation of IRS-1. Tyrosine phosphorylation of the IRS proteins activates a complex signal transduction pathway involving several downstream molecules, including PI3K and Akt. Many studies have demonstrated the critical role of PI3K in insulin-stimulated glucose uptake and translocation of glucose transporter-4 (GLUT4) to the cell membrane [33]. The two main pathways that regulate GLUT4-mediated glucose uptake are the PI3K/Akt and AMPK signaling pathways. To clarify the mechanisms by which saponins, including compound **6**, enhance glucose uptake, we determined the phosphorylation states of PI3K, Akt, and AMPK in insulin-resistant HepG2 cells. These experiments revealed that compound **6** activated both the PI3K/Akt- and AMPK signaling pathways in these cell lines (Figure 4 and Figure 5).

Further studies are needed to determine the detailed mechanisms underlying the effects of compound **6** on glucose metabolism, especially its effects in animal models of diabetes mellitus. Studies should also examine the molecular mechanisms by which saponins regulate the AMPK signaling pathway and, the effects of compound **6** on plasma insulin levels, for example. Nevertheless, the present study is the first to demonstrate that saponins from *S. chinensis* DC. enhance glucose uptake and improve insulin sensitivity. Compound 6 extracted from *S. chinensis* DC. may be a particularly useful candidate compound for lowering blood glucose and improving insulin resistance in the treatment of T2DM.

## 4. Materials and Methods

### 4.1. Materials

The HepG2 cell line was purchased from the Chinese Type Culture Collection (Shanghai Institute of Cell Biology, Chinese Academy of Science, Shanghai, China). MTT and insulin were purchased from BioSharp (Hefei, China). Dulbecco’s modified Eagle’s medium (DMEM) and fetal bovine serum (FBS) were purchased from Thermo Fisher Scientific (Waltham, MA, USA). A glucose assay (glucose oxidase method) was purchased from Beijing Kinghawk Pharmaceutical Co., Ltd. (Beijing, China). The assay kits for HK and PK were purchased from Nanjing Jiancheng Bioengineering Institute (Nanjing, China). The cell lysis buffer containing phosphatase inhibitor and proteinase inhibitor was purchased from Roche (Basel, Switzerland). Anti-IR and anti-IRS-1 antibodies (anti-Rabbit, 1:1000) were from Epitomics (Burlingame, CA, USA). Anti-AMPK, anti-p-AMPK, and p-IRS-1 antibodies (anti-Rabbit, 1:1000) were from Cell Signaling Technology (Beverly, MA, USA). Anti-PI3K, anti-p-PI3K, anti-Akt, anti-p-Akt (anti-Goat, 1:100) were from Santa Cruz Biotechnology (Santa Cruz, CA, USA). Anti-β-actin antibody (anti-Mouse, 1:1000) was from Abgent (San Diego, CA, USA). Western blotting images were processed using a ChemiDoc XRS digital imaging system with Multi-Analyst software (Bio-Rad Laboratories, Inc., Hercules, CA, USA).

### 4.2. Cell Culture and Induction of Insulin-Resistant HepG2 Cells (HepG2/IR)

HepG2 cells were maintained in low-glucose DMEM (Hylcone, Thermo Fisher Scientific, Waltham, MA, USA) supplemented with 10% FBS (Hylcone, Thermo Fisher Scientific, Waltham, MA, USA) 100 units/mL penicillin, and 100 μg/mL streptomycin (BioSharp, Hefei, China) and were kept at 37 °C in a humidified atmosphere of 5% CO_2_ in air. To induce insulin resistance, the cells were seeded onto six-well plates at 3 × 10^5^ cells/well or in 96-well plates at (4 × 10^3^ cells/well) for 24 h until they reached 80% confluence. Cells were then serum-starved for 24 h. After pretreatment for 24 h in serum-free DMEM with normal (5.5 mm) or high (30 mm) concentrations of d-glucose, cultured in 100 mg/L insulin for 24 h, cells were harvested and tested as described below.

### 4.3. Cell Viability Assay

HepG2 cells were seeded onto 96-well plates at a density of 4 × 10^3^ cells/well for 12 h. HepG2 cells were pretreated with 5–40 µm of the indicated compound dissolved in DMSO (final concentration of DMSO: 0.1%). The cytotoxicities of the test compounds were determined by incubating cells in MTT to assess viability. Cell viability was calculated by dividing the absorbance of cells exposed to a test compound by the absorbance of control cells exposed to the vehicle (DMSO) alone.

### 4.4. Glucose Uptake

Insulin resistance was induced in HepG2 cells as previously described [26] with minor modifications. Before the experiments, the cells were resuspended by 0.25% trypsin and replated onto 96-well plates. After the cells reached 80% confluence, the medium was replaced with serum-free DMEM. After 12 h, the cells were cultured in high-glucose DMEM supplemented with 2% fetal calf serum (FCS) and 100 mg/L insulin (BioSharp, Hefei, China) for 24 h. Six wells were exposed to high-glucose DMEM supplemented with 2% FCS without insulin. After stimulation with insulin for 24 h, the cells were washed four times in serum-free, high-glucose DMEM and twice in “phosphate-buffered saline (PBS). Cells were then cultured in serum-free, high-glucose DMEM with or without the indicated compounds at the specified concentrations or a positive control (metformin, 10 μm) for 24 h. After 24 h, the glucose concentration in the culture medium was measured using the glucose assay kit (glucose oxidase method). The glucose uptake rate was calculated by subtracting the glucose concentration at the end of the culture period from the glucose concentration of the high-glucose DMEM. To take into account cell proliferation/viability, glucose uptake was assessed by dividing the glucose uptake rate by the MTT absorbance.

### 4.5. Assays for HK and PK Activities

Cells were cultured in six-well plates at a density of 3 × 10^5^ cells/well. After reaching 80% confluence, the cells were treated as described for the glucose uptake assay. Briefly, human HepG2 cells were treated without or with 100 mg/L insulin for 24 h, and were washed four times with serum-free high-glucose DMEM and twice with PBS. The cells were then incubated in serum-free high-glucose DMEM containing the test compound or metformin (10 μm) for 24 h. After 24 h, the cells were washed three times with PBS, harvested in PBS at 4 °C, and mechanically disrupted with a Selecta Sonopuls (HD 200, Bandelin, Germany). The HK and PK activities were measured using HK and PK assay kits (Nanjing Jiancheng, Nanjing, China)

### 4.6. Western Blotting

Cells were seeded into six-well plates (3 × 10^5^ cells/well) for 12 h and then serum-starved for 12 h. After pretreatment, cells were treated with high-glucose DMEM supplement with 2% FCS and 100 mg/L insulin for 24 h. Cells were then treated without or with the test compound, and washed four times in serum-free high-glucose DMEM and twice in PBS. After 24 h, cells were washed three times with PBS at 4 °C, and harvested in lysis buffer containing phosphatase and proteinase inhibitor. The nuclear fraction was discarded after centrifuging cells at 10,000 revolutions/min for 10 min. Aliquots of cellular lysates (50 μg protein) were separated by 10% sodium dodecyl sulfate-polyacrylamide gel electrophoresis (SDS-PAGE) for 8% SDS-PAGE and IRS-1, or 6% SDS-PAGE for p-IRS-1(Ser 307). The separated proteins were transferred to nitrocellulose membranes (Pall Corporation, Washington, NY, USA). The membrane was blocked in 5% skimmed milk (BioSharp, Hefei, China) for 1 h, then incubated with the primary antibody (anti-IR, anti-AMPK, anti-IRS-1, anti-p-IRS-1, anti-PI3K, anti-p-PI3K, anti-Akt, anti-p-Akt, and anti-β-actin) overnight at 4 °C, and then incubated with an appropriate secondary antibody (Abbkine, Redlands, CA, USA) for 1 h at room temperature. Blots were developed using an enhanced chemiluminescence western blot detection system (Beijing, China) and the immunoreactive bands were visualized and measured by densitometry, with the ChemiDoc XRS digital imaging system and Multi-Analyst software (Bio-Rad, Hercules, CA, USA)

### 4.7. Statistics and Graphics

All data are expressed as the mean ± the standard deviation (SD) of three independent experiments. GraphPad Prism software (GraphPad, San Diego, CA, USA) were used for graphic representation and statistical analysis. Statistical significance was determined using analysis of variance with Tukey’s *post* *hoc* test. Values of *p* < 0.05 were considered statistically significant.

## 5. Conclusions

In conclusion, administration of compound **6**, a triterpenoid saponin isolated from *S. chinensis*, significantly enhanced glucose uptake and activated HK and PK, thus ameliorating insulin resistance, in HepG2 cells with insulin resistance induced by high glucose and insulin. These effects of compound **6** were mediated by the AMPK and IR/IRS-1/PI3K/Akt signaling pathways in insulin-resistant HepG2 cells. Taken together, our results suggest that *S. chinensis* is a candidate herbal medicine for treating T2DM.
